# Thrombotic microangiopathy during carfilzomib use: case series in Singapore

**DOI:** 10.1038/bcj.2016.62

**Published:** 2016-07-29

**Authors:** Y Chen, M Ooi, S F Lim, A Lin, J Lee, C Nagarajan, C Phipps, Y S Lee, N F Grigoropoulos, Z Lao, S Surendran, E M Teh, Y T Goh, W J Chng, S K Gopalakrishnan

**Affiliations:** 1 Department of Haematology, Singapore General Hospital, Singapore; 2 Department of Haematology-Oncology, National University Cancer Institute, Singapore

Carfilzomib is an irreversible proteasome inhibitor and is an effective treatment for multiple myeloma (MM).^1, 2^ It received US Food and Drug Administration approval based on a single-arm multicenter trial of carfilzomib monotherapy in 266 patients with relapsed MM following at least two prior lines of treatment including bortezomib and immunomodulators.^1, 3^ Safety analyses from four phase II carfilzomib trials (*N*=526) that included heavily pre-treated MM patients suggested a favorable risk-benefit profile with no specific signal of thrombotic microangiopathy (TMA) reported. However, serious adverse events of anemia, thrombocytopenia and increased serum creatinine comprised 1.3%, 1.1% and 1.3%, respectively.^4^ Over 350 subjects have been enrolled in randomized phase III trials in Asian countries. Herein, we report four cases of TMA related to carfilzomib use among 24 patients from 2 tertiary hospitals in Singapore (Table 1). All patients who started carfilzomib had a creatinine clearance >30 ml/min and platelet counts>50 × 10^9^/l at the start of treatment.

Patients 1 (70/Chinese/Male) and 2 (66/Chinese/Female) had newly diagnosed MM and were treated in an institutional review board-approved investigator-initiated study (IIS) using carfilzomib in combination with cyclophosphamide and dexamethasone as frontline therapy for high-risk MM (SGHMM1, NCT02217163). Within this trial, carfilzomib is given at 20 mg/m^2^ in cycle (C) 1, days (D) 1 and 2 followed by 56 mg/m^2^ for all subsequent doses as tolerated. Patient 1 had reported fever and flu-like symptoms on C2D2. The carfilzomib dose on C2D2 was postponed to C2D3 after fever resolved. He developed grade 1 diarrhea the day after, and on C2D6 hemoglobin declined from 7.5 to 5.3 g/dl and platelets dropped from 105 × 10^9^ to 5 × 10^9^/l. This was accompanied by emergence of schistocytes on the blood film and an acute rise in serum creatinine (209 μmol/l from 97 μmol/l). Hemolytic screen was positive (LDH 1833 U/l, bilirubin 24 μmol/l, reticulocyte index 2.6, haptoglobin<0.1 g/l) with a negative Coomb's reaction. Prothrombin time (PT) was 11.7 s and activated partial thromboplastin time (APTT) was 27.2 s. The diagnosis of TMA was made, and carfilzomib was discontinued. Rhinovirus was tested positive from throat swabs, stool cultures were negative and ADAMTS13 activity was normal. Platelet counts and renal function recovered to baseline 4 days after diagnosis of TMA and cessation of carfilzomib.

Patient 2 presented on C2D8 with symptoms of anemia and a dry cough. Investigations showed Hb 6.1 g/dl (from 9 g/dl), WCC 2.88 × 10^9^/l and platelets 55 × 10^9^/l (from 351 × 10^9^/l) with schistocytes seen on blood film and positive hemolytic screen. Acute kidney injury was noted with a rise in serum creatinine from 93 to 573 μmol/l. The patient had no evidence of infection and ADAMTS13 activity was 88% and there was no coagulopathy. The last dose of carfilzomib was on C2D2 and no further carfilzomib was administered. The patient required temporary hemodialysis but not plasmapheresis. Her platelet counts recovered after 7 days and renal function normalized after 1 month.

Patient 3 (63/Chinese/Male) had a 10-year history of MM and had previously received multiple lines of therapy including VAD (vincristine, doxorubicin and dexamethasone), high-dose melphalan (HDM) with autologous hematopoietic stem cell transplant (HSCT), bortezomib and immunomodulatory agents. Carfilzomib 27 mg/m^2^ and dexamethasone were commenced. Blood counts at treatment initiation were: Hb 10.5 g/dl, WCC 3.13 × 10^9^/l and platelets 224 × 10^9^/l. On C2D15, he presented with fever, diarrhea, cough and tested positive for parainfluenza B virus. Three days later, he developed acute kidney injury (creatinine 403 μmol/l from 76 μmol/l) accompanied by thrombocytopenia (platelets 3 × 10^9^/l) and non-immune hemolytic anemia. There was no coagulopathy (PT 9.9s, APTT 34.0s). Stool was negative for *Escherichia coli*. Carfilzomib was held off and he was expectantly monitored. He did not require plasma exchange. Platelet counts recovered to baseline after 25 days and renal function recovered after 60 days.

Patient 4 (58/Chinese/Male) had a 2-year history of MM and had received induction with bortezomib, cyclophosphamide, dexamethasone followed by HDM and autologous HSCT. He had relapsed within 12 months of HDM, while receiving lenalidomide maintenance and was given two cycles of bortezomib-DCEP (velcade, dexamethasone, cyclophosphamide, etoposide and cisplatin) as salvage therapy. His MM progressed despite treatment and he started carfilzomib 27 mg/m^2^ and dexamethasone. Prior to initiation of carfilzomib, serum creatinine was 277 μmol/l, Hb 9.1 g/dl, WCC 6.62 × 10^9^/l and platelets 92 × 10^9^/l. He tolerated the first two cycles well but was admitted on C3D7 with fever of 3-day duration and decreased urine output. Investigations revealed Hb 5.9 g/dl, WCC 5.67 × 10^9^/l, platelets 15 × 10^9^/l, creatinine 1133 μmol/l, PT 10.5s and APTT 27.1s. Schistocytes were seen on blood film accompanied by positive hemolysis markers. Stool cultures were negative. Patient was initiated on hemodialysis. There was platelet recovery after 10 days. At the time of this report, this patient's renal function is yet to improve. Patients 3 and 4 received carfilzomib on a named patient program (NPP). All four patients were treated with bortezomib following resolution of TMA without further complications. Patients were not rechallenged with carfilzomib.

We present 4 cases of TMA in 24 patients treated with carfilzomib giving a cumulative incidence of 16.7% (Table 1). While there have been a few single reports associating carfilzomib use with TMA,^5, 6, 7^ our case series is the largest reported to date. Two of our patients had newly diagnosed MM and received carfilzomib as frontline therapy. TMA occurred at 56 mg/m^2^ dose for the two patients in the IIS and at 27 mg/m^2^ dose for the two patients on the NPP. The dose regimens of 56 mg/m^2^ were chosen for the IIS based on previous phase I/II studies which demonstrated tolerability.^8, 9, 10^ Events that led to treatment discontinuation in the 20 and 56 mg/m^2^ regimens that have been previously published include decline in cardiac function, fever and infection. There was one event of TMA reported that did not result in treatment discontinuation.^7^ Of these four patients, one patient required plasmapheresis while the other three were managed expectantly. Two of the patients had concurrent viral URTI. Hypertension and proteinuria, considered the hallmarks of VEGF inhibition and a possible mechanism of drug-related TMA, were absent.^5, 11^ Among single cases of TMA due to carfilzomib reported in the literature, one achieved stable renal function after cessation of carfilzomib,^5^ another discontinued carfilzomib and received plasmapheresis with renal recovery^6^ and the third patient had improvement in TMA after dose reduction of carfilzomib.^7^

Carfilzomib is a potent agent in treatment of MM as randomized studies have shown and it is increasingly being used for treatment in both frontline and relapse settings.^2, 12^ We observed some unique features in the case series which will help clinicians. The incidence of TMA in the two Singapore centers was higher than previously reported. All of our patients developed TMA in the first three cycles. All four patients were of Chinese descent. We note a similar presentation of fever, diarrhea and URTI symptoms that preceded the development of TMA in three patients. None of the patients developed extra-renal manifestations of TMA such as stroke or other arterial events. With drug cessation and supportive care, and hemodialysis in one patient, the TMA was self-limiting in all cases. Finally, all these patients could be treated with bortezomib after resolution of TMA.

We believe concurrent cytotoxic agents, coexisting viral infections and patient characteristics contributed to the higher incidence of carfilzomib-associated TMA in our population. Further genetic studies are being planned as there seems to be a higher incidence of TMA among our predominantly Chinese patients treated at the two centers. Fever and diarrhea preceded the onset of TMA in our patients and may be useful early clinical markers. While drug dosages continue to be refined in different population groups and mechanisms leading to development of TMA associated with carfilzomib continue to be investigated, we suggest vigilance when carfilzomib is used, and consideration of early cessation when TMA is suspected.

## Figures and Tables

**Table 1:** Carfilzomib regime in patients with carfilzomib-related TMA

*Carfilzomib regime*	*No. of patients treated with regime at two institutions*	*No. who developed TMA*
*Endeavor trial Carfilzomib arm (Cd)*		
Cycle 1 D1, 2—IV 20 mg/m^2^ Cycle 1 D8, 9, 15, 16 and subsequent cycles D1, 2, 8, 9, 15, 16 (of 28 day cycles)—IV 56 mg/m^2^	11	0
*Carfil-Cyclo-Dex IIS (CCd)*		
Cycle 1 D1, 2—IV 20 mg/m^2^ Cycle 1 D8, 9, 15, 16 and subsequent cycles D1, 2, 8, 9, 15, 16 (of 28 day cycles)—IV 56 mg/m^2^	10	2
*Named patient program (Cd)*		
Cycle 1 D1, 2, 8, 9, 15, 16 (of 28 day cycle)—IV 20 mg/m^2^ Subsequent cycles D1, 2, 8, 9, 15, 16 (of 28 day cycles)—IV 27 mg/m^2^	3	2

## References

[bib1] Khan ML, Stewart AK. Carfilzomib: a novel second-generation proteasome inhibitor. Future Oncol. 2011; 7: 607–12. Available from http://www.futuremedicine.com/doi/abs/10.2217/fon.11.42. 21568676 10.2217/fon.11.42 PMC3931449

[bib2] Stewart AK, Rajkumar SV, Dimopoulos MA, Masszi T, Špička I, Oriol A et al. Carfilzomib, lenalidomide, and dexamethasone for relapsed multiple myeloma. N Engl J Med 2015; 372: 142–152. 25482145 10.1056/NEJMoa1411321

[bib3] Herndon TM, Deisseroth A, Kaminskas E, Kane RC, Koti KM, Rothmann MD et al. US Food and Drug Administration approval: carfilzomib for the treatment of multiple myeloma. Clin Cancer Res 2013; 19: 4559–4563. 23775332 10.1158/1078-0432.CCR-13-0755

[bib4] Siegel D, Martin T, Nooka A, Harvey RD, Vij R, Niesvizky R et al. Integrated safety profile of single-agent carfilzomib: experience from 526 patients enrolled in 4 phase II clinical studies. Haematologica 2013; 98: 1753–1761. 23935022 10.3324/haematol.2013.089334 PMC3815177

[bib5] Hobeika L, Self SE, Velez JCQ. Renal thrombotic microangiopathy and podocytopathy associated with the use of carfilzomib in a patient with multiple myeloma. BMC Nephrol 2014; 15: 156. 25267524 10.1186/1471-2369-15-156 PMC4190298

[bib6] Lodhi A, Kumar A, Saqlain MU, Suneja M. Thrombotic microangiopathy associated with proteasome inhibitors. Clin Kidney J 2015; 8: 632–636. 26413293 10.1093/ckj/sfv059 PMC4581378

[bib7] Lendvai N, Hilden P, Devlin S, Landau H, Hassoun H, Lesokhin AM et al. A phase 2 single-center study of carfilzomib 56 mg/m2 with or without low-dose dexamethasone in relapsed multiple myeloma. Blood 2014; 124: 899–906. 24963043 10.1182/blood-2014-02-556308 PMC4624439

[bib8] O'Connor OA, Stewart AK, Vallone M, Molineaux CJ, Kunkel LA, Gerecitano JF et al. A phase 1 dose escalation study of the safety and pharmacokinetics of the novel proteasome inhibitor carfilzomib (PR-171) in patients with hematologic malignancies. Clin Cancer Res 2009; 15: 7085–7091. 19903785 10.1158/1078-0432.CCR-09-0822 PMC4019989

[bib9] Alsina M, Trudel S, Furman RR, Rosen PJ, O'Connor OA, Comenzo RL et al. A phase I single-agent study of twice-weekly consecutive-day dosing of the proteasome inhibitor carfilzomib in patients with relapsed or refractory multiple myeloma or lymphoma. Clin Cancer Res 2012; 18: 4830–4840. 22761464 10.1158/1078-0432.CCR-11-3007

[bib10] Moreau P, Kolb B, Attal M, Caillot D, Benboubker L, Tiab M et al. Phase 1/2 study of carfilzomib plus melphalan and prednisone in patients aged over 65 years with newly diagnosed multiple myeloma. Blood 2015; 125: 3100–3104. 25784682 10.1182/blood-2015-02-626168

[bib11] Eremina V, Jefferson JA, Kowalewska J, Hochster H, Haas M, Weisstuch J et al. VEGF inhibition and renal thrombotic microangiopathy. N Engl J Med 2008; 358: 1129–1136. 18337603 10.1056/NEJMoa0707330 PMC3030578

[bib12] Dimopoulos MA, Moreau P, Palumbo A, Joshua D, Pour L, Hájek R et al. Carfilzomib and dexamethasone versus bortezomib and dexamethasone for patients with relapsed or refractory multiple myeloma (ENDEAVOR): a randomised, phase 3, open-label, multicentre study. Lancet Oncol 2016; 17: 27–38. 26671818 10.1016/S1470-2045(15)00464-7

